# Book Reviews

**Published:** 1812-10

**Authors:** 


### CRITICAL ANALYSIS
### OF RECENT PUBLICATIONS
IN THE
### DIFFERENT BRANCHES OF PHYSIC, SURGERY, AND MEDICAL PHILOSOPHY.

---

*History of the Royal Society from its Institution to the End of the Eighteenth Century. By* THOMAS THOMSON, M. D. F.R.S. L. and E. &c. 4to. London, 1812. pp. 552.—lxxxiii.

THE influence which the Royal Society has had in promoting the progress of medical knowledge, and the improvement of the sciences connected with it, as well as the numerous dissertations on all these subjects contained in the Philosophical Transactions, makes it our duty to notice Dr. Thomson's History of that Institution; a volume which has afforded us both amusement and instruction.

The author arranges his work under an historical introduction, five books, and an appendix: of each of these divisions we shall attempt a short analysis.

Much of the success of human projects depends on fortuitous circumstances, probably not noticed by those who form the first plans. To the fortunate coincidences existing when the Royal Society was projected, its rapid progress may, in great measure, be attributed; but to the character, abilities, and ardor, of its original members, must be referred its early reputation. Like many other stupendous fabrics, the Royal Society began with views that, perhaps, did not extend beyond the private or social gratification of those who composed its original meetings: but, the first associates being persons of considerable ability, and employed on subjects of high interest, the privacy of their meetings did not prevent their fame going abroad.

"About the year 1645, several ingenious men, who resided in London, and were interested in the progress of mathematics and natural philosophy, agreed to meet once a-week to discourse upon subjects connected with these sciences. The meetings were held sometimes in Dr. Goddard's lodgings, in Wood-street, because he kept in his house an operator for grinding glasses for telescopes; sometimes in Cheapside; and sometimes in Gresham College. In the year 1648 and 1649, several gentlemen who attended these meetings being appointed to situations in the university of Oxford, they instituted a similar society in that city, in conjunction with several eminent men already established there. Among the primitive members of this Oxonian Society, ciety, we find the following celebrated names: Dr. Wilkins, Dr. Wallis, Dr. Goddard, Dr. Seth Ward, Dr. Bathurst, Dr. William Petty, Dr. Willis. The meetings were held at Dr. Petty's lodgings; and, when that gentleman went to Ireland in 1652, the society met first in Dr. Wilkins's apartments, and afterwards in those of Mr. Bogle.

"The greatest part of these Oxford gentlemen, coming to London in 1659, held their meetings twice a-week at Gresham College; on Wednesday, after the astronomical lecture of Mr. Christopher Wren, and on Thursday, when Mr. Laurence Rooke lectured on geometry. Here they were joined by several new associates; among others by Lord Brouncker, W. Brereton, esq. Sir Paul Neile, John Evelyn, esq. Thomas Henshaw, esq. Henry Slingesley, esq. Dr. Timothy Clark, Dr. Ent, William Balle, esq. Abraham Hill, esq. Dr. William Croune. These meetings were continued till the members were scattered by the disasters of 1659, after the resignation of Richard Cromwell, when their place of meeting was converted into quarters for soldiers. But, after the restoration of Charles II. in 1660, these meetings were revived, and still more numerously attended. On the 28th of November 1660, a number of gentlemen met in Mr. Rooke's apartment, Gresham College, and agreed to constitute themselves into a society for the promotion of all kinds of experimental philosophy. A set of regulations were drawn up, a weekly contribution of a shilling was collected from each of the members, in order to defray the expenses of those experimental investigations. At first the number of members was limited to 55, but it was afterwards extended, and, finally, admission was left open to every proper candidate. A president, a secretary, and a register, were elected out of the body, and an amanuensis and operator hired to execute the purposes of the society."

Such was the origin of the Royal Society. In July 1662, Charles the 2d granted a royal charter, constituting them a body corporate, under the name of the Royal Society, appointing Lord Viscount Brouncker the first president, and Dr. Wilkins and Mr. Oldenburg, the first secretaries. The first meetings of the society after its incorporation were held at Gresham College; but, in 1667, Mr. Henry Howard, afterwards duke of Norfolk, presented to it the Arundel library, with apartments in Arundel-house. The society afterwards returned to Gresham College in 1673; but, at length, purchased a house in Crane-court, Fleet-street, where the society continued to meet until furnished with apartments in Somerset House.

The Philosophical Transactions, the result of the labors of the Royal Society, were at first published by the secretary in small numbers, consisting of a sheet or two, either monthly or seldomer, as materials were supplied. Mr. Oldenburgh, the first secretary, began the Transactions. The first number was published in 1665; and he continued the publication till the period of his death 1677, in all 136 numbers, making nearly the whole of the first twelve volumes. He was succeeded by Dr. Nehemiah Grew, who was chosen secretary on the 30th of November, 1677: he published number 137, and continued editor for two years. The last number which he published was 142. Dr. Grew was succeeded by Dr. Hooke, but the publication of the Trans. was intermitted for three years. In 1681, Dr. Hooke began to publish what he called *Philosophical Collections;* which have always been considered as constituting a portion of the Transactions, though under a different name. Dr. Hooke continued his publication of the Collections during the years 1681 and 1682, and published in all seven numbers. But in 1683 Dr. Plot, who had, the preceding year, been chosen secretary in place of Dr. Hooke, undertook to revive the publication, on condition that the society would bind themselves to purchase 60 copies of each number. Dr. Plot was editor during 1683 and 1684, and published the 13th and 14th volumes, comprehending all the numbers between 143 and 166. The 15th volume, from number 167 to 178 inclusive, was edited by Mr. William Musgrave, who was secretary during 1685.

This volume, probably, did not give satisfaction to the society. There seems also to have been a deficiency of materials, for Dr. Halley, in 1686, offered, on condition that the publication should be renewed, to furnish one fourth of the whole out of his own private stock. It appears that Dr. Halley was editor of the 16th volume, comprehending the numbers between 179 and 191 inclusive, and published during the years 1686 and 1687. After the publication of this volume there was an interval of three years without the appearance of any thing more; owing obviously to the deficiency of materials. The publication was again revived in 1691; and, though Dr. Halley was not the ostensible editor, he appears to have been actively concerned in superintending the publication till the period of his voyage to the southern hemisphere in 1698: for there is an order of council, passed about that time, enjoined Dr. Tyson, Mr. Hart, Dr. Sloane, Mr. Waller, and Dr. Hooke, to assist Dr. Halley in drawing up the Transactions. Volumes 17 and 18, consisting of the numbers between 192 and 214, and published during 1691, 1692, 1693, and 1694, were ostensibly edited by Mr. Waller, who had been elected secretary in November 1687. The ostensible editor of the volumes from 19 to 28 inclusive, between 1695 and 1713, was Sir Hans Sloane, who had been chosen secretary in November 1693. Dr. Halley, chosen secretary in 1713, was editor of the 29th and 30th volumes, published published between 1714 and 1719, at the rate of two numbers a-year. The 31st, 32d, 33d, and 34th, volumes, published between 1720 and 1727, at the rate of two numbers annually, were edited by Dr. James Irvin, chosen secretary at the end of 1721. Dr. Rutty, chosen secretary 1727, was editor of the 35th volume, consisting of 8 numbers, published in 1728. Dr. Cromwell Mortimer, elected secretary in 1730, for twenty years edited and published 11 volumes, beginning with the 36th. The 46th volume was published in 1750, and was the last that was published in numbers.

In 1750, a committee was appointed to superintend the publication; and the Transactions since that period have been published in half volumes. For the first twelve years, only one half volume was published annually; but, from 1762, two half volumes, or a complete volume, have appeared every year.

In this immense body of arts and sciences, down to the year 1800, is contained 4166 papers. Of these 107 are on Botany, 82 on Vegetable Physiology, 44 on Agriculture, 290 on Zoology, 131 on Anatomy, 90 on Comparative Anatomy, 220 on Physiology, 478 on Medicine and Surgery, 38 on Mineralogy, 251 on Geognosy, 29 on Mining, 67 on Geography and Topography, 208 on Mathematics, 416 on Astronomy, 137 on Optics, 40 on Dynamics, 120 on Hydronamics, 26 on Acoustics, 48 on Navigation, 211 on Electricity, 71 on Magnetism, 406 on Chemistry, 281 on Meteorology, 87 on Chemical Arts, 12 on Weights and Measures, 39 on Political Arithmetic, 120 on Antiquities, and 66 Miscellaneous.

Medicine, Astronomy, and Chemistry, are the sciences which furnish the greatest number of papers; but Electricity is that which is most completely developed.

It appears that the proportion of general merit in the Phil. Trans. has depended on the president for the time being. On this subject Dr. Thomson expresses himself in the following terms:

"The nature of the papers read before the Royal Society, and of course the value of the Transactions published, were considerably influenced by the president. If any person will take the trouble to examine the volumes published during the presidency of Martin Folkes, esq. and compare them with the rest of the work, he will find a much greater proportion of trifling and puerile papers than are any where else to be found. It was during this period that Sir John Hill published his *Review of the Works of the Royal Society,* in which he endeavors, with all the humor and all the knowledge he was master of, to throw ridicule upon the labors of that illustrious body. Immediately on the election of Lord Macclesfield, who succeeded

Martin

Martin Folkes as president, in 1752, a very striking improvement is observable in the value of the Transactions. Many excellent papers made their appearance during the presidentship of the Earl of Morton. The unfortunate dispute between Mr. Wilson and the other electricians of the Royal Society, about the relative goodness of pointed or knobbed conductors, occupies too great a proportion of the Transactions during the presidentship of Sir John Pringle, yet the volumes of that period contain many memorable papers: as, for example, Dr. Maskelyne's experiments at Schehallien to determine the density of the earth, with Dr Hutton's deductions from them; the experiments of Sir George Shuckburgh Evelyn and General Roy, in order to establish correct formulas for measuring heights by the barometer; the report of the committee appointed by the Royal Society to determine the proper method of graduating thermometers; the experiments of Mr. Hutchins to ascertain the freezing point of Mercury; and many others. Whoever will examine the Transactions with care, will easily satisfy himself that by far the most valuable volumes of that work are the thirty-two which have been published during the presidentship of Sir Joseph Banks, and, fortunately for the progress of science, he has enjoyed that situation for a much longer period than any of his predecessors."

These are the leading features of the history of the establishment and progress of the Royal Society, an institution to which science has been indebted in many essential points. Dr. Thomson's work, however, is principally occupied in giving a connected detail of the progress of knowledge, as contained in the Philosophical Transactions. With this view he arranges the materials of the Transactions under five heads or books: the first of these contains the Natural History part, and is subdivided into four chapters—1. Botany; 2. Zoology; 3. Mineralogy; 4. Geography and Topography.

In the first chapter of this book the author gives a slight sketch of the origin and progress of botany to the period of the establishment of the Royal Society, and then proceeds to examine the materials found in the Transactions on this subject.

"One of the declared objects of the Royal Society at its establishment was Botany. Botanical committees were established; directions given to those members who went abroad to send accounts of rare plants; correspondents were established in America, Asia, and Africa, for the same purpose; and persons were employed to traverse England in order to collect plants."

But, notwithstanding this comprehensive and well devised plan, it seems that, of the 107 botanical papers in the Phil. Trans. only 38 can be considered as of any value. Only 24 new species of plants are described. Several of the most eminent botanists, though Fellows of the Society, did not contribute contribute a single paper.* The advantages derived to the science of botany from the efforts of the Royal Society, were not to be looked for or expected in the papers contained in the Phil. Trans. but, as in many other instances, to the stimulus given to the spirit of inquiry. And our author observes "that it seems in some measure owing to these liberal and enlightened exertions, and to the subsequent labors of Ray, that the study of botany became extremely fashionable in England, and produced that cluster of eminent men whose labors Linnæus dignifies with the name of the golden age of botany." Of many of these eminent men, Dr. Thomson has here given biographical notices. How, Merret, Plukenet, Petiver, Sloane, Sherrard, Dillenius, Morison, Ray, Tournefort, and Linnæus, have each some attention given to them.

The second section of this chapter goes into a history of inquiries respecting the *Anatomy and Physiology of Plants*. "This branch of botany possesses more dignity than the preceding. It opens to our view some of those beautiful contrivances which Nature follows to accomplish her purposes, and which irresistibly lead her enlightened observer to the knowledge of the existence and benevolence of an all-powerful intelligent Being." To the Royal Society science is entirely indebted for the origin of this species of investigation. By the members of that society the inquiry was begun, and the first laborer in this field was Dr. Grew. Of Dr. Grew the following account is given:

"He was born in Coventry about 1628. His father, who was a non-conformist, sent him abroad at an early age; and he took the degree of Doctor of Medicine at a foreign university. After his return to England, he settled in London, became a fellow of the Medical College, and practised medicine with some degree of success, till the period of his death, 1711. He began to turn his attention to the anatomy of plants as early as 1664. In 1670, he put an essay on this subject, which constitutes the first book of his *Anatomy of Plants,* into the hands of his brother-in-law, Dr. Henry Sampson, who shewed it to Mr. Oldenberg, at that time secretary to the Royal Society. By Mr. Oldenberg it was given to Dr. Wilkins, bishop of Chester, by whom the manuscript was read to the Royal Society. That learned body highly approved of it, and ordered it to be

* Since the commencement of the Linnæan Society in 1788, no botanical papers have been admitted into the Transactions of the Royal Society. That this is an amenity due from the Royal to the Linnæan Society, few will dispute. The research and ability manifested in the 10 vols. of the Linnæan Society's Transactions, establish its claim on public opinion.

printed printed in 1671. At the suggestion of Dr. Wilkins, Dr. Grew was appointed Curator to the Royal Society for the Anatomy of Plants. This occasioned the drawing up of the 2d, 3d, and 4th, parts of his Anatomy of Plants; and of the various lectures on the same subject, which constitute a part of that work. The whole of these papers were written between 1670 and 1676; and read at intervals during various meetings of the Royal Society. The whole were collected in 1682, and published in a folio volume, by order of the Society. It constitutes a book well known by the name of *Grew's Anatomy of Plants;* a book which contains a great deal of valuable and important matter, and which has always been referred to as a classical work on the subject."

The labors of Malpighi, Dr. Stephen Hales, Duhamel, Hedwig, Gærtner, Bonnet, Senebier, Mirabel, and Knight, are also noticed. Of Gærtner, so well known for his elegant work *De Fructibus et Seminibus Plantarum,* the fullest account is given we have yet seen in an English work.

The propagation, fecundation, discovery of the sexual organs, and the structure of plants, is examined; as is also their functions, their food, ascent of the sap, transpiration, irritability, &c. From this part, did our limits permit, much might be extracted, with which we believe our readers would not be displeased.

The remaining portion of this chapter relates to agriculture and the œconomical uses of plants. It will be immediately seen that this section, as having but a remote connection with the subjects to which our publication is destined, need be little more than noticed by its title. Some parts, on the cultivation and propagation of *saffron,* it may, however, be proper to state.

"The culture of the *Crocus sativus* was introduced from the Low Countries. Some of the cakes consist of the styles and stigmas of the flowers, without any addition; but towards the end of the crop, when the flowers become small, it is customary to sprinkle the cake with a quantity of beer. On account of the high price of saffron, and the small quantity produced, *safflower* has been introduced by the apothecaries as a substitute, and is commonly sold instead of it."

By *safflower* we suppose the author means the flower of the *Carthamus Tinctorum,* and there is some reason to apprehend the foreign saffron is so sophisticated; but that, probably, not by the apothecaries, as is here asserted, but by the growers or manufacturers, before it comes into the hands of the medical practitioner. The Hon. Charles Howard, afterwards Duke of Norfolk, and Dr. James Douglass, have papers in the Phil. Trans. on the cultivation and preparation of of Saffron. The first is in vol. xii. p. 945, for 1678; the other in vol. xxxv. p. 566, for 1728.

The second chapter of the first book treats of *Zoology*, and in the comprehensive extent here given to it, very properly occupying nearly one-fifth of the volume. It is subdivided into five sections: the 1st treating of the Arrangement and Description of Animals; the 2d, of the Anatomy; 3d, of Comparative Anatomy; 4th, of Physiology; 5th, of Medicine and Surgery.

Intimately as this chapter is connected with the science of medicine, from entering minutely into the history of animal life, we are compelled, from obvious reasons, to give but a slight and imperfect sketch of it.

Following the plan he adopted in the first chapter, the author gives a rapid view of the progress of zoological knowledge, beginning with Aristotle, and proceeding to Pliny, Ælian, Aldrovandus, Johnston, Willughby, Ray, Ellis, Mouffet, Fabricius, Smeathman, Linnæus, and Pennant, of all of which, and their discoveries or arrangements, he gives short notices. Reckoning from the commencement of the Royal Society to the end of the year 1800, there are in its Transactions 290 papers on Zoology, applying to Mammalia, Birds, Amphibia, Fishes, Worms, and Insects, which contain much curious and interesting matter, though necessarily of various degrees of merit. Of these 290 papers, "there are 125, which seem in the present advanced state of the science, scarcely to be entitled to any attention whatever." Some have a mediocrity of merit, and "many are excellent." Dr. Thompson goes into a copious detail, which will be read with much satisfaction. The articles on the fascinating powers of serpents, on the fecundity of fish, the tyrian purple, aphides, the termites, and coccus, contain much interesting information.

The second section, treating of *Anatomy*, gives a moderately comprehensive view of the progress of this art among the ancients, comprehending the time of Hippocrates, to the foundation of the Royal Society; and then notices the great discoveries of the circulation, the lacteals, and lymphatics: in which the names of Vesalius, Harvey, Cowper, Douglass, Vieussens, Lower, Willis, Bartholine, Rudbeck, Winslow, Monro, Haller, &c. hold an important station, and are followed by an enumeration of 32 of what the author considers as the most important anatomical dissertations in the Transactions, with a short statement of their contents. To this succeeds the section treating of *Comparative Anatomy*.

This subject, which is daily rising in importance, occupied throughout every period much of the attention of the Royal

Society. The papers relative to it are very numerous in the Phil. Trans.; and in Dr. Thomson's work is enumerated 51 of the most interesting. The dissection and description of the structure of 43 different animals, will evince the ardor with which the fellows of the Royal Society and their friends applied to comparative anatomy.

The fourth section is upon a subject of peculiar interest—the properties and functions of living bodies, under the restricted term *Physiology*. From the time of Galen, and his book *De usu Partium*, the first treatise expressly on Physiology, a rapid glance is given of the progress of opinions; and our author acutely observes, that the principles of physiology have uniformly depended upon the particular science that happened to be in fashion.

"When the opinions of Aristotle and Galen reigned paramount in the schools, all the functions of living bodies were explained by having recourse to occult qualities and occult faculties. When Paracelsus drew the attention of Europe to chemistry, that science was considered as paramount to account for all the animal functions; and the physiologists of the time explained every thing by means of fermentation, sublimation, distillation, filtration, concoction, and other similar processes, familiar to the chemists of the age. When Newton and his contemporaries laid the foundation of mechanics upon the rigid principle of mathematical demonstration, physiologists embraced with eagerness the fashionable doctrines; the human body was converted into an hydraulic machine; the force of the heart and the velocity of the blood were rigidly ascertained; and every thing was accounted for by the size, and shape, and motion, of the different particles of matter of which the body was composed. When mechanical philosophy began to lose its novelty, it was in some measure supplanted as a fashionable study by a peculiar species of metaphysics, which was prosecuted with much ardor for a time, till it at length terminated in universal scepticism. During the progress of this enticing science, physiologists laid hold of its notions and doctrines, and two opposite systems were produced, the more ancient explaining every thing by the action of a *living principle*, and the more modern by a principle somewhat indefinite, to which they gave the name of *irritability*. The recent discoveries in pneumatic chemistry having again brought that bewitching science into fashion, a new race of physiologists have arisen, who ascribe every thing to chemical principles, and ring changes upon the words *oxygen, hydrogen, carbon*, and *azote;* by means of which, in their opinion, every function of the living body may be satisfactorily explained."

The opinions of Boerhaave, Hoffman, Stahl, Cullen, Brown, and Darwin, are cursorily noticed; but the palm is given to Haller.

"I consider," says Dr. Thomson, "the physiology of Haller to be be by far the most important work on the subject which has hitherto appeared. It is the most stupendous monument of industry which the eighteenth century produced. Instead of indulging, like most of his contemporaries and predecessors, in constructing an ingenious hypothesis to account for the functions of living bodies, Haller undertook the gigantic task of collecting all the *facts* relative to the subject, which had been ascertained. This he accomplished in his Elements of Physiology, which consists of eight pretty thick quarto volumes."

In imitation of this illustrious example, our author, instead of attempting an outline of the science, collects and arranges all the physiological facts which have appeared in the Phil. Trans. under the subsequent heads: 1. Circulation of the Blood. 2. Respiration. 3. Action of the Skin. 4. Nervous System. 5. Vision. 6. Organs of Motion. 7. Nourishment and Digestion. 8. Generation.

The facts particularly noticed under the first head are—quantity of the blood in animals, in which the experiments of Dr. Moulin, and the controversy between Keill and Jurin are examined; force and velocity of the blood, and particularly Hale's experiments in the second volume of his Statical Essays; nature of the blood, comprehending the notions of Leeuenhoek, and father Di Torre, with the experiments and opinions of Hewson and Darwin, transfusion of blood. This project, which was originally suggested by Sir Christopher Wren, and prosecuted by Lower and King, became at the time an object of great interest, and was eagerly adopted by the French physicians. Its total inutility, and the difficulties and hazards accompanying its employment, have sunk it, deservedly, into neglect.

Under the second head, *Respiration*, the mechanical part of this function, the effects of air, and the origin of animal heat, are detailed. On the last, the opinions of the ancients, the mechanical physicians, of Drs. Hales, Cromwell Mortimer, and Crawford, and the facts stated by the late Dr. Currie, of Liverpool, and very lately by Mr. Brodie, are noticed.

The *Action of the Skin*, making the third head, states particularly facts respecting the sensible and insensible perspiration, color of Negroes, and the capability of living bodies to bear a high temperature. Under the last is given an epitome of the experiments made by Dr. Fordyce and Sir Charles Blagden, in 1775.

The fourth head, treating of the *Nervous System*, enters into the inquiries and opinions respecting a nervous fluid; the re-union of cut nerves on the experiments of Dr. Haighton; the structure of the nerves; theories respecting sleep; instances of long-continued sleep; of torpor, and the hybernation of various animals; poisons, with the experiments of Messrs. Courten and Herissant; sensibility of diseased bones and tendons.

The fifth head, on *Vision*, details the opinions of Mariotte, his controversy with Pequet, and the highly-interesting history of the blind boy, related by Cheselden.

*Muscular Motion*, under the denomination, organs of motion, is the subject of the sixth head. In this the origin and views of the Croonian lecture are related; hypothetical causes of muscular motion; and experiments on the eye, the subject of the lecture intended to have been given by Mr. Hunter, but whose death deprived the world, probably, of the most ingenious disquisition that this Croonian project has produced. Annexed to this subdivision, is an account of a paper by Mr. Robertson, written to determine the specific gravity of living men.

The seventh head treats of *Nourishment;* relates the observations on the mechanism of digestion; attempts to explain that function; action of the bile; effects of madder on the bones; and remarkable instances of abstinence. Most of the hypothetical explanations of digestion have been discussed in the Phil. Trans., and we have here a neat epitome of those discussions.

The most inexplicable, as well as the most important, of animal functions is *Generation*, the subject of the eighth subdivision of this section. In this the doctrine of Harvey, the experiments of Needham and Buffon, the hypothesis of Leeuenhoek, and the discoveries of Haighton, are epitomized. Several other questions are likewise noticed, as the nourishment of the fœtus in utero; the crying of children in the womb; the peculiarity of the organs of generation in the kanguroo; the number of young produced at a birth by various animals; and of numerous births in the human female.

The fifth section of this chapter, on *Medicine and Surgery*, commences with an historical detail of medical systems and opinions, from Hippocrates to Darwin, of whose *Zoonomia* Dr. Thomson expresses himself with pointed severity.

"I consider," he says, "the theory of Darwin as merely the Brunonian doctrine under a disguised form, introduced with a greater parade of learning and science. The great number of new terms which it contains makes it a very irksome task to peruse it. Great pains are taken, throughout, to discard every appearance of a living principle, and to establish medical practice on the broad and vulgar basis of materialism."

The concluding observations of this historical sketch, are scarcely scarcely less severe on the science of medicine altogether. Perhaps, however, there is some ground for making them; and, if they irritate the profession into scientific precision, we shall readily forgive the slight character of petulance with which they are marked.

"Such is a short sketch of the history of medicine down to our own time. From a view of the whole we are intitled to conclude, that medicine, if we are to consider it as a science, has made very little progress since the days of Hippocrates. New theories, indeed, and new modes of explanation, have successively appeared in abundance; but they have been all equally visionary and absurd, and have vanished without leaving any traces behind them. If we attend to the opinions and writings of the medical men of the present day, we find a propensity among some to restore the exploded chemical doctrines of Paracelsus, amended, of course, and improved. Others lean pretty strongly to the humoral doctrines of the ancients, obviously in consequence of the strong bias which the popular systems of Hoffman and Cullen gave medicine, to direct the whole of the attention to the solids."

To medicine, viewed as an art, the author is a *little* more complaisant, and allows it to have "undergone great and progressive improvements."

"The materia medica," he adds, "has been gradually and almost entirely changed; and the present mode of treating patients, though *perhaps* not less efficacious than that of the ancients, is infinitely superior in every thing that concerns the feelings of the patient."

Of the 478 papers on medical subjects contained in the Phil. Trans., only a few of the prominent articles are here noticed, under 22 heads or paragraphs. Of these, we can do little more than enumerate the subjects. The 1st is an analysis of Sydenham's work on Fevers; the 2d is on Cantharides; the 3d, on Small-pox; the 4th, on cold Water in Fevers; 5th, on Spontaneous Combustion, of which several instances are given; 6th, on the Jail Distemper; 7th, on Dropsy; 8th, on Blisters in Fever with Cough; 9th, Effects of Camphor; 10th, Capt. Cook's Method of preserving the Health of Seamen; 11th, Case of Nephrotomy; 12th, Case of wounded Trachea; 13th, Inoculation for Small-pox; 14th, on the Origin and Introduction of Lues; 15th, Palliative Treatment of Calculus; 16th, Cortex in Mortification; 17th, Cure of Ascites by Injection; 18th, Improvement in Lithotomy; 19th, Extraction of the Crystalline Lens; 20th, Case of Deafness; 21st, on the Regeneration of Bones; 22d, on the Cure of wounded Intestines.

Among these articles some will be found of much greater importance than others; we particularly recommend, to the attention of our readers, numbers 5, 6, 10, 14, 17, and 21.

The third chapter, on *Mineralogy*, though containing much interesting information, as being but remotely allied to the science of medicine, will claim less attention than the preceding. It is divided into three sections, the first of which, under the title of *Oryctognary*, gives a view of the systems of Linnæus, Cronstedt, Werner, and Haüy; and extracts from the Phil. Trans. some account of the Fullers'-earth Pits in Bedfordshire; the discovery of Diamonds in Brazil; Tourmaline; native Tin, and native Lead.

The second section, of *Geognosy*, treats of Petrifactions in general; Vegetable Petrifactions; Bovey Coal; petrified Wood at Lough Neagh; of Shells and Zoophites; of Bones; of Mosses, and the Irruption of Solway Moss; of Earthquakes, and their Cause; of Volcanoes, and Hot Springs, particularly the Temperature of the Bath Waters; of the Deluge, with Woodward's, Halley's, and King's, Theories; of the Height of Mountains; Changes in the Earth's Surface; the supposed Isthmus between Britain and France; Coral Islands; Formation of Marble; and Descriptions of Places: concluding with a sketch of the *Wernerian Geognosy*.

The third section treats of *Mining*, in which we meet with an account of the British mining districts; of the mines of Transylvania and Hungary; the salt mines of Poland and Cheshire; the tin mines of Cornwall; and the diamond mines of India; with a relation of the precipitation of copper by *iron*.

The fourth chapter, on *Geography* and *Topography*, is circumstanced like the third, with respect to medical science. It treats of the Latitude and Longitude of Places; the Construction of Maps; Hydrology; and Topography. In the latter division is some account of the North-American Indians, and the Patagonians; with observations on the Falkland Islands, and Thibet.

We must here take leave of Dr. Thomson's work so far as it relates to Natural History; the remaining part, above half the volume, applies to sciences, in which, with one exception, chemistry, the physician and the naturalist will find but few facts to illustrate their studies.

The second book, on *Mathematics*, pursuing the same course with the preceding, gives an historical detail of the progress of this branch of science, down to the period of the Royal Society; connecting preceding discoveries with the establishment of that institution, and epitomizing the materials furnished by the Philosophical Transactions.

The third book, on *Mechanical Philosophy*, is divided into nine chapters, treating on Astronomy, Optics, Dynamics, Mechanics, Hydronamics, Acoustics, Navigation, Electricity, city, and Magnetism. This book must afford considerable interest, as relating to many of the important discoveries of Sir Isaac Newton.

In the fourth book, which gives the history, theory, and practice, of *Chemistry*, the connection with medical science is more obvious than in the second and third. The subject is divided into three chapters: Chemistry, strictly so called; Meteorology; and Chemical Arts and Manufactures. As in the preceding parts, the science is deduced from its origin, and pursued in its progress down to the present time. The alchemists gave birth to the science of chemistry; Paracelsus, Van Helmont, Agricola, and Libavius, partly allied to these fanatics, pushed on the investigation, and were succeeded by Boyle, Hooke, Newton, Becker, Stahl, Black, Cavendish, Priestley, Bergman, Scheele, Lavoisier, Bertholet, Fourcroy, and Morveau; all of whom are here noticed. From these Dr. Thomson proceeds to, what he calls, the new British chemists, or those who succeeded to the English chemists who had given up the phlogistic hypothesis and the science together. Of the new British chemist the following account is given:

"By degrees, a new race of chemists arose, founded at first, as one may say, on the French school, and moulded after their model. Dr. George Pearson led the way by his translation of the French Nomenclature, and by the various valuable papers which he published in the Philosophical Transactions. By degrees the reverence, almost approaching to absurdity, which was paid to the French school, wore off, and the natural genius and invention of British philosophers began to appear. It would be scarcely consistent with propriety to particularize the discoveries which have been the result of this happy revolution, and which have restored Great Britain to the first rank among the improvers of chemistry which she formerly held, as the celebrated men to whom we are indebted for them are still alive, and actively engaged in extending the boundaries of the science. But we may mention that three distinct schools (if we may use the expression) have been established by three gentlemen, certainly not the least celebrated among British chemists. These are, Dr. Wollaston, who possesses an uncommon neatness of hand, and who has invented a very ingenious method of determining the properties and constituents of very minute quantities of matter. This is attended with several very great advantages; it requires but little apparatus, and therefore the experiments may be performed in almost any situation; it saves a great deal of time and a great deal of expense; while the numerous discoveries made by Dr. Wollaston demonstrate the precision of which his method is susceptible. The second of those schools has been established by Mr. Davy (now Sir Humphrey Davy), whose galvanic discoveries and analyses of the alkalies have constituted a new era in the science, and furnished with with a totally new method of experimenting. Mr. Davy is the most active, as well as the most celebrated, chemist of Great Britain, and many more discoveries may be expected from him. The third school has been established by Mr. Dalton, who has not yet obtained that degree of celebrity in this country to which he is entitled, and which he will infallibly acquire. His atomic theory is one of the greatest steps that the science has hitherto made; and it appears to be established by the most indisputable evidence."

The chemical papers in the Phil. Trans., down to 1800, amount to 406. Our author passes over 118 of these as useless or trifling; of the remainder, he gives some notices under the following arrangement.

1st. *Gases.* "The papers on this subject in the Phil. Trans. are curious in an historical point of view. We see the first rude notions of these bodies gradually improving, till this branch of the science acquired the perfection and beauty which it has now reached." In the thirty papers on the gases, which are published in the Phil. Trans., many curious facts are related. The *damps* of mines had particular attention given to them. They are described as of four kinds by Mr. Jessop. 1. The common (carbonic acid gas). 2. The pea's blossom, so called from its smell, and concerning the nature of which nothing accurate is known. 3. The bag; this is described as a visible round ball, hanging at the top of the mine, which, if it be broken, disperses itself, and suffocates all the miners. We know nothing about its nature. 4. The fire damp, which is found to be carburetted hydrogen. The experiments of Boyle, Huygens, Papin, Hauksbee, Greenwood, Sir James Lowther, Maud, and Clayton, are stated. Mr. John Maud, in 1736, collected the gas which escapes during the solution of iron filings in diluted sulphuric acid, and exhibited its combustion before the Royal Society; and this, perhaps, was the first attempt in England to make experiments upon pure hydrogen gas. Of the experiments of Hales, Brownrig, Cavendish, Preistley, Fontana, Ingenhousz, Darwin, Milner, Tennant, Pearson, and Henry, the results are given, and the volumes of the Transactions containing the details are referred to.—2d. *New Substances.* The Phil. Trans. contains among its papers the original descriptions of many of these substances. Twenty-seven of the most remarkable of these are enumerated by Dr. Thomson. —The 3d division of this arrangement treats of the invention of new and important instruments, and new methods, under the title of *Apparatus.*—The 4th is on *Analyses*, and contains particularly the earliest attempts, as being most curious in historical facts.—The 5th, under the term *Processes*, contains a number of curious facts, of which our limits prevent a detail. The names of Goddard, Lister,

Lister, Jackson, Mitchell, Watt, Blagden, Beddoes, and Franklin, will mark the importance of this part.—The 6th is on *New Experiments*. From the multitude of these, which cannot be reduced to any general head, the author selects some which he deems most to deserve attention. We observe amongst them, methods for producing artificial cold, for inflaming oils by nitric acid, for determining the boiling point of water, and Mr. Kirwan's experiments on sulphurated hydrogen gas.

The second Chapter of this book we cannot but consider to be on a subject of importance to the medical practitioner: it relates to changes in the atmosphere, under the title of *Meteorology*. The weight of the atmosphere, temperature, evaporation, rain, dew, winds, and aurora borealis, are the meteorological circumstances particularly examined. An account is given of the *Harmattan*, an extraordinary wind on the west coast of Africa, between the equator and north latitude 15°, which blows several times a-year from the NE. from five to fifteen days together. The peculiarities of this wind we shall relate at some future opportunity. The relative quantities of dew remaining upon a variety of bodies is shewn in a table by Dr. Stocke.

The third Chapter, *on Chemical Arts and Manufactures*, contains the methods of making vinegar in France, white lead, alum, copperas, sal ammoniac, Salop, Tokay wine, &c.

The fifth Book, consisting of *Miscellaneous Articles*, has four chapters. The 1st is on Weights and Measures; the 2d is on political Arithmetic, from which we might extract, had we room, observations on the healthiness of London, and on the population of Great Britain; the 3d is on Antiquities; and the 4th contains subjects not arrangeable under other heads.

We have now gone through a work which, as before observed, has afforded us much entertainment, and not a small portion of information. That it contains more than the title promises, will hardly be considered as a fault by any purchaser. The wide field on which the author enters, shews his ardor in the undertaking; the scientific researches point out the extent of his acquirements; the clearness of the arrangement decides on his capability to render complex subjects simple and perspicuous. We can wish, however, that he had added to these excellencies a language more polished and copious. But we have, notwithstanding, seldom met with a book of the same extent containing such abundance of useful materials, exciting so much interest, and affording, with so little alloy, such an exuberance of scientific gratification.

*The Principles of Physiological and Physical Science, comprehending the Ends for which Animated Beings were created, and an Examination of the Unnatural and Artificial Systems of Philosophy which now prevail. By* RICHARD SAUMAREZ, ESQ. 8vo. pp. 425. Murray, Egerton, &c. &c.

IT is impossible, with the limited means which we possess, to give a complete detail of the various and important subjects which are contained in this work; we, nevertheless, feel it due to our readers, to lay before them some of the new principles of philosophy which it developes.

In the first Chapter,—*On the Principles of Science*,—Although the author adopts the Baconian system of induction, he reprobates the misapplication of it in physiology and in physics, he says, "instead of appealing to simple observation for the apprehension of natural phenomena, few *natural* phenomena are admitted to be true, unless proved by the test of experiment; unnatural effects are generally preferred to those which are natural and unsophisticated; the phenomena of disease are adduced to explain the actions of health, the chemical changes, which dead and common matter undergo, are assumed, in order to account for the cause and phenomena of life." After illustrating these assertions, by facts, the author observes, "in the analysis of facts, which are intended to constitute the principles of any science, none should be admitted but such as are scientifically efficient of the conclusion; so that the effect produced shall always correspond to the nature of the producing cause; we should separate partial from general facts, accidental and transient attributes from those that are permanent and essential. It is by a process, such as this, that we become possessed of those individual facts, which form the base and the source of every science, the immediate and approximate cause whence effects are derived. Without a full possession of these permanent and universal facts, a general, not a particular knowledge of any subject can ever be obtained; without history we can never have definition, and without axiom there can be no science." p. 11. These observations are so just and self-evident, that it were greatly to be wished they were more attended to than they are at this time.

In the second Chapter, *on the essential Properties of Matter with Relation to Vitality*, after separating the essential from the secondary or accidental properties of matter, Mr. Saumarez reprobates the classification employed of the material world into animal, vegetable, and mineral, as being defective.

fective. Instead of comprehending the whole, it excludes a part: it excludes that immense and indefinite portion of matter, which, instead of being immured in the bowels of the earth, subsists for the most part out of it, water, and gaseous bodies in general, and the whole planetary system in particular. He therefore classes the whole under the three distinct heads of *living*, of *dead*, and of *common*, matter. 1. By living matter, he comprehends the various orders of living beings with which the universe is replenished and adorned. 2. By dead matter, he confines himself to the exuviæ of animals and vegetables, as well as to the whole substance of which the system is composed, after the actions of life are at an end, and the state at present known by the appellation of death. 3. By common matter, he means the primitive or original materials or elements of which the world is composed; matter which either has never received the participation of life, or, having received, has lost it, and been resolved back into a common state.

As this classification comprehends all the objects which Nature embraces, it at once gets rid of the objection above stated. The author then proceeds to remark the great and striking difference which subsists between each. He observes, "if we contemplate the nature of *common matter*, at rest or in motion, at unity or in union; whether the changes it undergoes proceed from motion mechanical, from mixture chemical, or from both together; we shall find it an universal truth that the same effects universally result from the operation of the same causes. In *living beings* it is far otherwise; when the assimilating organs perform their functions with force and with efficacy, they possess the power of changing and destroying the sensible and chemical qualities of the substances they receive; they not only possess the power to act, but to resist action—to change things external to themselves, without being changed by external things—to convert them, instead of being converted *by* them. The commutation which the food has undergone is total and complete; gasses are bereaved of their expansibility, alkalies of their acrimony, all of the order of their affinities, and rendered bland and mild; solids are liquified, liquids gelatinized and made solid; things inanimate are animated; animated things are killed and revived; the most sapid bodies are rendered insipid; the most putrid matter is deprived of its putridity and made fresh, the most fresh is made susceptible of undergoing the processes of putrefaction and fermentation. The matter, therefore, which every living system receives for its nourishment and support, can only arise out of its *aptitude*, and its aptitude proceed from its imbecility and weakness. It is as inert as the shoe is without the foot, or as the musical

 instrument instrument without the power of the musician; it is to the energy of a living power (which by some has been called a germinating principle) resident in the seeds of plants, and in the fecundated ova of animals, that the acorn becomes evolved into an oak, and the process of nutrition and of growth carried on. It is this power which constitutes the architect, by which the machine is erected; it is the base on which the whole stands, it forms the bond of its elementary parts, the cement which unites them in one whole; it is the cause, primary and efficient, from whence the individuality of every system arises, in which the form and the sex it assumes essentially reside; by which the human species differs from the brute, the brute from the vegetable, the vegetable itself from matter inanimate and common. It is this power which is called *life*, and the matter which this power has assimilated and organised, what he calls *living* matter. The author therefore defines the principle of life to be, *that power by whose energy different species of matter are assimilated to one kind, a living system, organized and formed, and the various parts of which it is composed are protected or preserved from decomposition or decay!*

The third, fourth, fifth, and sixth, Chapters are on the *Nature* of the Principle of Life and of Mind, on Sensation and its objects, and on the Physiology of *organic* Life, comprehending the means by which organic actions are excited and supported, suspended and destroyed, and the whole of the system decomposed into its elementary parts. Although there is much new matter in these chapters, and old facts put in a new point of view, we are under the necessity of referring the *physiological* reader to them, in order to arrive at what may be called the *physical* part of the work.

The seventh Chapter is *on the Elementary Properties of Matter*. The author begins by saying, "that, after having detailed the state of deprivation in which matter, in general, exists with relation to the principle of life, it will be easy to comprehend the nature of its existence after it has lost the participation of life which it had received, and after it has been finally resolved back to its common state, whether of a fluid or of a solid kind. Instead of contemplating the nature of extension and of bulk, which matter in general possesses, by virtue of which it has the capacity or aptitude to be acted upon by the agency of external means, we rather search for the properties which external means have produced upon it, and, instead of separating, confound *figure* and *extension* together."

After shewing the state of original deprivation of all secondary qualities in solid matter, Mr. Saumarez says that, it is equally equally referable to liquid also, to that element which may be considered as constituting the principal source and support of fluidity in general. If water possessed flavor, together with fluidity, it would, in such a case, be the flavor of the water that was tasted, and not the flavor of the substance which the water was intended to dissolve and to support. If water was salt or sour, bitter or sweet, sapid instead of being altogether insipid, those secondary qualities would constantly manifest themselves, and the quality of the receiver would always adulterate and obliterate the quality of the thing received."

After pursuing the same train of reasoning at some length, he observes, "it is the same with air and light: air bears the same relation to sound that water does to flavor, or that the block does to figure. If air possessed any particular sound of its own, that sound, like the figures upon the canvas, would manifest itself; either when it was impelled through musical instruments in general, or the vocal organs in particular; instead of harmony, discord would be produced. Air, however, possessing the primary and essential attributes of expansibility alone, it has the power to expand, and the capacity to be compressed; to be modulated and harmonized by the energy resident in the organs of speech; and, by their instrumentality, language is produced."

After shewing that air is destitute of solidity and of color, he proceeds, with the same principles, in order to shew that pure elementary light, with respect to color, subsists in a negative state also; that, if the medium of air through which the rays of light are conveyed, instead of being diaphanous and permeable, were turbid and colored, that the light would be arrested and constantly tinged and dyed by the colors of the medium, as it is when the rays pass through glasses stained with different colors, when the color of the glass is always found to stain the rays of light, and convey to the eye its particular color; through a green glass the object looks green, through a red glass it looks red, &c.

M. Saumarez observes, "If the colored state of the medium through which objects are beheld produces these unnatural consequences, how much more must these unnatural consequences be produced, if the rays of light are themselves colored, originally and essentially! If, in fact, the light which proceeds from the sun to the earth consists, as Sir I. Newton asserted, of seven colors, which are original and simple, and that all the colors in the world either consist of these simple colors, or of compounds formed out of them, mixed together in different proportions."

After combating this hypothesis, the author proceeds to prove, prove, by the universal fact of the increasing degree of coldness at high degrees of altitude from the surface, in all latitudes, from the equator to the poles, that the sun is not a globe of fire. "We are therefore," he observes, "driven to the necessity of concluding, notwithstanding the mixture and opacity of the medium in which we exist, is involved, that there subsists in it rays of light that are neither hot nor cold, fire nor ice, yellow nor green, &c. &c. but that are *transparent* and *colorless* only; that are as colorless as air is speechless, as much as water is tasteless, or as solid matter is motionless; but that are destitute of every essential and original quality, *extension* and *motion* alone excepted. Consequently, it may be presumed that the sun itself, as the parent whence the rays of pure light proceed, is a globe of light only, &c. &c."

The second section of this chapter describes the mechanical power of light, and the various experiments that have been made on the solar rays by Herschel, Woollaston, &c. in which it appears that it is Dr. Herschel to whom the merit unquestionably belongs of having separated the *element* from the *accident*, the pure colorless light from the compound and visible color. On the whole, M. Saumarez concludes by observing, "that the phenomena which light describes, decidedly prove that it is of a quality *sui generis*. Instead of being solid, massy, and impenetrable, it is the most fluid, the most subtile, and the most penetrable, matter, that can be conceived*; that, instead of being inert and heavy, it is essentially active and imponderable; instead of moving, like the matter of our earth, by a power from without, the solar rays traverse with the most incredible velocity the wide expanse of the planetary spheres, exciting the sensation of illumination on the optic organs of animated beings, by a power from within, inherent and essential."

With respect to the spots in the sun, the author observes, "it may be presumed, that the various observations which have been made by Dr. Herschel and others, on the nature of the dark and opake matter, of which the body of the sun is supposed to be composed, are optical errors, from the insufficiency of the instruments employed; that these spots, instead of constituting different integrant portions of the sun itself, are rather to be considered as collected masses of opake matter, interposed in the medium alone; that from the fugitive and evanescent nature of these spots, it is reasonable to suppose, that they exist below the disc of the sun, and are out of it; that they are totally unconnected with it, and form no part of its body." The immense distance of the sun, the necessity of blackening the lens through which the rays pass, pass, and the confession of Dr. Herschel himself, that the means we possess of reaching it are insufficient, will, we trust, excuse the author of presumption in drawing such a conclusion.

He proceeds, in the eighth chapter, to investigate the means by which compound bodies are formed. The effect produced is ascribed to chemical affinity or elective attraction; and he takes occasion to combat the Newtonian idea of the solidity and impenetrability of matter. "So far from solidity or impenetrability being essential properties of matter, we possess facts, abundant and universal, to shew that the primary particles of matter are both permeable and penetrable; in proof of it the author adduces, 1. The conversion of the different kinds of food which animated beings receive for their nourishment and support, by the process of assimilation from a dead to a living state, from insensibility to sensibility: without the penetrability of matter, no such change could be accomplished. 2. The change total and complete which takes place between different portions of matter, by chemical combination, and more especially by combustion, &c., he conceives, prove, that the penetrability of matter rests upon evidence as demonstrable as the attribute of extension itself.

The ninth chapter treats of the process of Gassification. We do not think that we should be doing justice to the author, in the brief analysis we are giving of his work, if we were to pursue in this place the arrangement which he has adopted; the subject that follows the matter of pure elementary and colorless light, ought evidently to be the means by which color is produced, which is contained in chapter xvi, *On Colorification in general.*

After having detailed the defective state of the analytic and synthetic arts, he observes, "Although a multitude of compound bodies can factitiously be formed from those that are simple, no effort of human ingenuity has been able to discover the means by which the pure and colorless solar rays, in their passage through the atmosphere, acquire properties totally different from those they originally possessed, and have the power, in consequence, of exciting on the optic organs of animated beings, the sensations of illumination in general, and of color in particular."

He proceeds to examine the Newtonian hypothesis, contained in the Lectiones Opticæ, in which it is assumed that the sun is a globe of fire, emitting rays, composed of seven different colors, which are separable from each other by the prism, and that to the difference of capacity, in different bodies, of absorbing particular rays and of reflecting others, the variety riety of colors in different bodies is supposed to be owing, that a red body, for example, reflects from its surface the red rays, and absorbs the rest; a green body reflects the green rays, and absorbs the rest; that a *white* body is supposed to reflect all the rays, and to absorb none; and a black body to absorb all the rays, and to reflect none. If the assertions were true, the author observes, "that the color of a body depended on the reflection of particular rays, and in the absorption of the rest; if a body be red, or green, or yellow, by the reflection of these individual rays, and the absorbtion of the rest, it must evidently follow from these assumptions, that the body which we suppose to be red, or green, or yellow, is, *ipso facto*, inherently and absolutely of every other color but red, or green, or yellow. We must conclude from thence, that the body which we call WHITE, because it reflects all the rays and absorbs none, is destitute of color altogether; and on the contrary, that the body which is known by the name of BLACK, because it absorbs all the rays and reflects none, is absolutely and inherently of every color but a black one. Absorbing all the rays and reflecting none, as the black body is supposed actually to do, it is impossible that any knowledge could be obtained by the eye of the figure and color of the body, in which all the colors were immersed. The same consequences would ensue with respect to what is called WHITE. If a body appear to be *white*, because it *reflects* all the rays and absorbs none of them, it is impossible that any knowledge could ever be obtained of a *white* body by the eye, since, in such a case, it must be destitute of color altogether. He asks, whether there be any solid ground for supposing, that the different impressions made on our organs of vision by the particular rays which flow from particular bodies, are not equally referable to them all, without any exception. Whether any exception ought to be made to jet and to snow; whether the rays that flow from both, by which the impressions on our optic sense excite the sensations of *black* and of *white*, have not an actual existence on the surfaces of those bodies, as much as on those from gold and indigo? from the white wax and black wick of a candle, as from the red flame that issues out of it? and whether that hypothesis is built upon a legitimate assumption, which makes the body which is called *black*, to arise from the absorption of all the rays, and at the same time affirms the substance, which reflects all the rays, and absprbs none, to be the cause of *white*, making in truth and in fact *black* to be *white* and *white* to be *black*.

After ridiculing with success the absurdities to which

 these these assumptions would lead, he observes, that they have been occasioned by an ignorance of physiology; by not observing the relation which exists between the *sensitive* principle *within*, and the *substance without*, by which sensation is excited and produced. Instead of ascribing sensation to the receiver, it has rather been referred to the thing received, and impression and sensation have thereby been confounded together.

After combating, at considerable length, the present doctrine of colors, Mr. Saumarez gives his own, the outline of which we shall present to our readers. "Instead of supposing that the pure solar rays, (which, in their simple uncombined state, he conceives to be colorless and invisible,) are colored originally and essentially; or that the infinite variety of colors which we behold, are formed out of the prismatic alone, it is far more reasonable to conclude, that the *pure* solar rays are the agents only; that not only the formation, but the diversity of color principally depends on the quality of the *base* with which colorless light has combined; that it is owing to this combination which has taken place, that a new substance is formed; which is colored since it is visible, and which is visible because it is combined; the properties of which are very different in their combined, from what they are in their simple elementary, state. The matter of light, from being colorless, becomes colored; from being transparent only, it participates, in an eminent degree, in the opacity and quality of the base, with which it has united, the result of which is the production of color. So long, therefore, as the eye becomes illuminated by any object whatever, that object must be considered to be colored. *Illumination* constituting the *genus* of which *color* is the *species*."

The remainder of this interesting chapter is occupied in illustrating by facts the truth of the hypothesis above stated; in the course of which the author observes, "No proof, perhaps, is more strong of the uniformity existing in the composition of the atmospheric matter, by which the medium above is filled, than the uniformity in the rays of color which are formed by the passage of the rays of light through it, and which are separable from each other by means of this prism, and thence called *prismatic colors*. It is for the purpose of describing the different mechanical phenomena which these different colors display, that the science of optics is especially designed, the leading proportions of which are afterwards detailed."

(To be continued.)

